# PMA-VQA: Progressive Multi-Scale Feature Fusion with Spatially Adaptive Attention for Remote Sensing Visual Question Answering

**DOI:** 10.3390/s26082351

**Published:** 2026-04-10

**Authors:** Yifei He, Chen Qiu, Jinguang Gu

**Affiliations:** 1 School of Computer Science and Technology, Wuhan University of Science and Technology, Wuhan 430065, China; hhhyfyyff123456@wust.edu.cn (Y.H.); simon@wust.edu.cn (J.G.); 2 Hubei Province Key Laboratory of Intelligent Information Processing and Real-Time Industrial System, Wuhan 430065, China

**Keywords:** remote sensing visual question answering, spatially adaptive attention, progressive multi-scale fusion

## Abstract

Remote sensing visual question answering (RS-VQA) is essential to intelligent Earth observation, as it supports interactive querying of high-resolution aerial images. Many existing methods struggle with fine-detail geospatial reasoning with remote sensing (RS) scenes due to RS scenes having intrinsic multi-scale object variance and pronounced spatial heterogeneity. The models tend to rely more on the linguistic prior than reasoning based on visual evidence. In this paper, we present PMA-VQA, a progressive multi-scale feature fusion with spatially adaptive attention, to embed the RS-VQA task in spatially based hierarchical feature integration. For hierarchical, multi-level, language-informed integration, we propose a spatial attention aggregation module (SAAM) and a progressive feature fusion and classification module (PFCM). The SAAM employs spatially adaptive gating to align cross-modal features with semantic context, while the PFCM integrates multi-scale representations across high-level semantic abstractions and low-level space. The experimental results on RS-VQA LR and HR benchmarks validate that PMA-VQA outperformed all competing methods in terms of accuracy and robustness. Evaluation of HRVQA further confirmed the effectiveness of the SAAM and PFCM across diverse RS scenes.

## 1. Introduction

Remote sensing visual question answering (RS-VQA) seeks to derive accurate responses to queries regarding overhead imagery. This differs from RS scene segmentation [1,2] and object detection [3,4], which focus on identifying and delineating specific entities. RS-VQA requires higher-level cognitive synergy between scene semantics and the intricate relational interactions of diverse geographical features [5]. This capability is essential for advancing automated systems in decision making in disaster response, environmental surveillance, and precision agriculture.

Early RS-VQA methods predominantly relied on fusion-based frameworks, in which vision and text encoders process images and queries independently, followed by cross-modal integration via mechanisms such as point-wise multiplication [6], global–local contextual attention fusion [7], or attention-based reasoning [8]. These methods have improved understanding at the occupancy level and relation modeling. Most recently, vision–language models (VLMs) [9,10] have been integrated for multitask RS-VQA by means of instruction fine-tuning for the purpose of aligning visual constituents with query tokens to produce answers. These models do exhibit impressive zero-shot potential, but they also have numerous weaknesses in the context of RS. Most of these models suffer from a language prior bias and respond based on the text rather than the visual evidence [11]. Furthermore, they also lack the requisite scale sensitivity and spatial understanding of satellite images.

**Figure 1:**
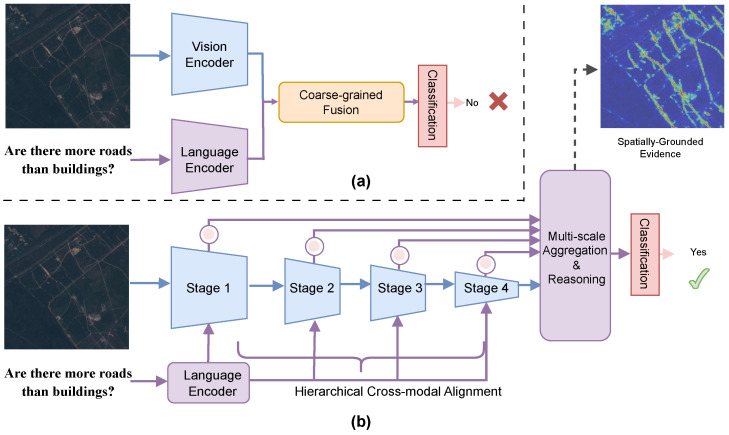
A comparison of conventional RS-VQA and our proposed PMA-VQA framework. (**a**) Current cross-modal fusion architectures adopt decoupled visual and linguistic encoders for coarse-grained alignment. (**b**) Our PMA-VQA implements a hierarchical fusion strategy to reconcile high-level semantic abstraction with fine-grained geospatial details toward RS-VQA.

The recent advancements in fusion-based models [6] and vision–language models (VLMs) [9,10] have been promising, but precise spatial-semantic grounding for multi-level, multi-scale RS imagery continues to be a challenge. Figure 1a shows that traditional structures adopt decoupled encoders, whereby visual and linguistic modalities are merged at a late stage and through coarse-grained fusion. Such approaches often fall victim to “language prior bias”, producing responses that reflect textual statistical regularities without any corresponding visual reality. As a result, and as exemplified by the question, “Are there more roads than buildings?”, these models often fail to capture fine-grained geospatial details and therefore are inaccurate in their detections.

Geographic regions with high spatial variability give rise to challenges associated with the diversity of semantic richness, requiring adaptive fusion at multiple levels. However, current methods apply global, uniform fusion weights over the entire feature map, rendering them insensitive to local semantic variations. In addition, small objects such as vehicles or buildings can only be reliably identified through fine-grained spatial details, whereas an exclusive reliance on high-level features inevitably discards the low-level spatial information necessary for accurate localization.

For these reasons, we formulate PMA-VQA, an approach that is capable of progressive multi-scale feature fusion and spatially adaptive attention (Figure 1b). We move from late-stage integration to a hierarchical cross-modal alignment approach. By infusing linguistic information into the four Transformer stages of the visual backbone, the model keeps steady semantic attention during feature extraction. This process facilitates unification of the high-level semantic abstractions with the fine-grained spatial cues required for resolving small-scale targets.

We introduce PMA-VQA, which is based on a Multimodal Swin Transformer architecture and enhanced with two core components, the spatial attention aggregation module (SAAM), and the progressive feature fusion and classification module (PFCM). More specifically, the PFCM is constructed to integrate multi-scale representations through a combination of spatial cross-alignment and a tiered convolution approach. This allows the model to provide spatially discriminative features for fine-grained localization tasks, such as the detection of ships or small buildings. Simultaneously, the SAAM employs a language-bound gating mechanism to assign location-aware importance, which lessens the spatial diversity that is inherent to RS scenes, i.e., the stark differences between dense urban areas and sparse rural areas.

The main contributions of this research are summarized as follows:

- We propose PMA-VQA, a framework incorporating a multi-level language guidance mechanism that enables hierarchical reasoning across visual stages, significantly reducing dependency on linguistic priors.
- We propose a spatially adaptive attention mechanism where the model deviates from typical uniform fusion and self-adjusts the recalibration of weights according to the local semantic density. A progressive multi-scale aggregation module is developed to harmonize high-level semantic abstractions with fine-grained spatial details.
- We validate PMA-VQA on three benchmarks spanning distinct resolutions, annotation regimes, and geographic domains. Comprehensive ablation studies isolate the functional contributions of the SAAM and PFCM.

## 2. Related Work

### 2.1. Fusion-Based Remote Sensing VQA

RS-VQA has emerged as an important multimodal framework for working with Earth observation (EO) data, as it has been designed to tackle the intricate geospatial dependencies and multi-scale variabilities of remote sensing images [6]. The preceding studies in this area have mainly focused on coarse-grained cross-modal relations in which task-specific encoders are merged with attention mechanisms to present integrated, language-attentive features. Illustratively, MAIN [8] introduced bilinear pooling architectures to effectively establish a mapping between localized spatial coordinates and textual semantic tokens. Hierarchical frameworks such as EarthVQANet [12] have been developed to address the extensive scale variance in RS scenes and combine the macro-level context of the scenes with micro-level detailed regional features. Focusing on more than just static architectures, EasyToHard [13] adopted a curriculum-based learning strategy that steers the optimization process from basic object recognition to advanced reasoning about spatial relationships. Recent advancements such as HFIF [14] and the SAMFPN [7] have further expanded the feature interaction schemes. HFIF exploits multi-level feature interplay to fine-tune cross-modal alignments, and the SAMFPN exploits the zero-shot abilities of the segment anything model (SAM) in a pyramidal structure to enhance scale-aware instance localization.

Nevertheless, fusion-based systems suffer from a “shallow interaction” bottleneck. Visual and linguistic streams are treated as separate streams, and cross-modal fusion is postponed to the final decision-making layers. Although recent work such as ACR [15] alleviates this by conditioning the fusion path on question semantics through type-guided cross-attention, the linguistic signal still does not modulate early-stage visual feature extraction. Such unidirectional or delayed interactions hinder linguistic queries from fluidly adjusting the feature extraction at the early stages. This frequently results in the model misidentifying the precise spatial entities that are being queried.

### 2.2. Vision–Language Models for RS-VQA

RS-VQA has recently moved to a new flexible approach based on vision–language models (VLMs) which seamlessly incorporate cross-modal reasoning with satellite images. RSGPT [16] initiated this change by adapting the InstructBLIP architecture to remote sensing domain specific feature alignment. Subsequent models like GeoChat [10] and Rs-llava [9] integrate multimodal instruction tuning for regional and image-level unified reasoning. SkyEyeGPT [17] significantly improves a model’s capacity for detailed questions by merging numerous vision–language tasks using a large-scale instruction-following dataset. DDFAV [18] reduces hallucinations and verifies fact consistency within the specific bounds of Earth observation.

Even following this progress, many current VLMs still struggle with a form of language prior bias, replacing pixel-level grounding with statistical patterns of language. This limitation is even more significant in remote sensing (RS) cases involving extreme spatial variability, where identical spectral signatures correspond to different land cover classes, depending on the environment [19]. Consequently, current models often lack the “spatial-semantic resolution” required to distinguish dense urban fabrics from sparse peri-urban landscapes, especially when distracted by inherent language biases.

### 2.3. Multi-Scale Reasoning and Spatial Grounding

More recent strategies for remote sensing visual question answering have begun concentrating more on multi-level reasoning and scene complexity with fine-grained visual verification. LPVA [20] proposes an adaptive hierarchical weighting mechanism for topological consistency during the feature encoding stage. TGFNet [21] builds upon sensor-induced interference and strengthens the architecture using the cross-modal consistency of the optical and SAR datasets. Tosato et al. [22] supported that the intricate spatial interplay between a discrete target and its geographical environment is the key point for localization accuracy. Complementary architectural insights have also emerged from the remote sensing change detection literature. SCAFNet [23] addresses the semantic misalignment between heterogeneous CNN and Transformer feature streams through a cross-attention-based semantic compensation module (SCM), an adaptive fusion module (CTFAF) that dynamically recalibrates local–global feature contributions via learnable parameterization, and a change feature identification module (CFIM) that suppresses pseudo-changes through channel-spatial reweighting. Unlike SCAFNet, which recalibrates representations within a single-modality Siamese encoder, our SAAM performs cross-modal alignment by computing question-conditioned gating weights at every backbone stage, enabling linguistic intent to actively modulate visual feature extraction throughout the hierarchy.

In contrast to existing methods that treat linguistic guidance as post hoc processing, PMA-VQA introduces a deeply coupled cross-modal strategy. Equipped with spatial-adaptive gating in the backbone, our framework allows linguistic guidance to actively control visual representation learning at each network stage. This structure is essential for the global comprehension and contextual significance of object identification in understanding RS scenes.

## 3. Methodology

### 3.1. Overall Framework

RS imagery displays multi-scale objects and spatial variability by nature. Addressing these challenges, we construct the PMA-VQA framework, which combines progressive multi-scale feature aggregation with a spatially adaptive attention mechanism. PMA-VQA supports *stage-by-stage* cross-modal feature extraction from high-level semantic abstractions down to fine-grained spatial details.

**Figure 2:**
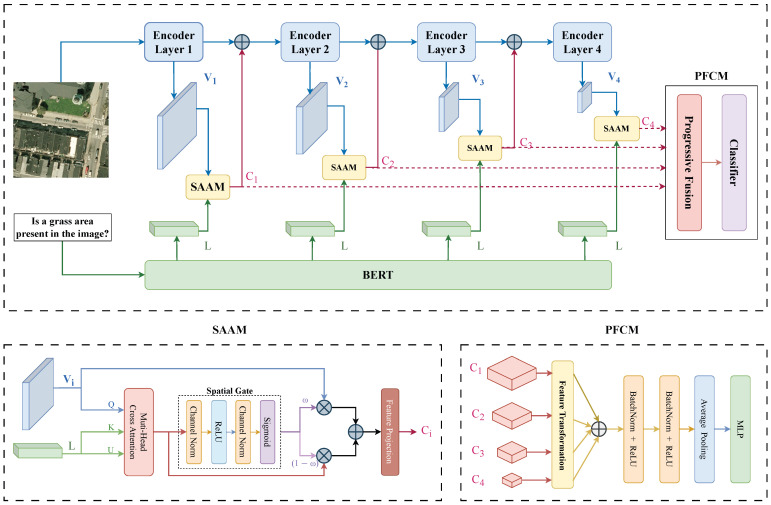
Overview of the proposed PMA-VQA framework for RS-VQA. The dual-stream pipeline extracts hierarchical visual representations via a Multimodal Swin Transformer and linguistic embeddings via BERT. A spatial attention aggregation module (SAAM) performs language-guided spatially adaptive gating with per-position fusion weights, where ⊗ denotes element-wise multiplication of the gated visual and linguistic streams and ⊕ denotes their element-wise addition. A progressive feature fusion and classification module (PFCM) consolidates all four scale levels for answer prediction.

As depicted in Figure 2, the system architecture follows a hierarchical dual-branch pipeline. The visual stream leverages a Multimodal Swin Transformer to extract a four-level feature pyramid ${\left\{V_{i}\right\}}_{i=1}^{4}$, and the linguistic stream encodes the input question through a pretrained BERT model [24] into a shared representation L. The interaction between these streams is governed by two key components. The first is the spatial attention aggregation module (SAAM), which performs language-guided spatially adaptive gating at all backbone stages and adjusts the cross-modal fusion weights according to the local semantic density. Although all four instances share the same linguistic input L, each learns independent projection and gating parameters for resolution-adaptive cross-modal fusion. The second is the progressive feature fusion and classification module (PFCM), which progressively fuses multi-level representations, from low-level detailed spatial information to high-level semantic abstractions, to tackle extreme scale variation. The entire architecture is optimized end-to-end via a multi-class cross-entropy objective. The notations and descriptions in this section are shown in Table 1.

### 3.2. Visual Encoder

To accommodate the multi-scale nature of RS objects, the visual encoder is constructed upon a hierarchical Swin Transformer [25]. Given an input RS image $I\in R^{3\times H\times W}$, it is first partitioned into P×P non-overlapping patches. These patches are projected into a *d*-dimensional latent space to form initial visual tokens:

(1) $$Z_{0}=F_{\mathrm{embed}}\left(I\right),$$

where $Z_{0}\in R^{N_{0}\times d}$ is the input to the first stage, $N_{0}=\frac{H}{P}\times \frac{W}{P}$ is the number of tokens, and $F_{\mathrm{embed}}\left(\cdot \right)$ is the patch embedding function with a dropout layer. Spatial position information is captured by a learnable relative position bias within each window attention module, rather than by an explicit absolute position embedding.

The backbone consists of four stages, where each stage has successive Swin Transformer blocks. Within each block *l*, the computational flow is defined as follows:

(2) $$\begin{array}{cc} {\hat{Z}}_{l}^{\left(i\right)} & =\mathrm{DropPath}\left(W-\mathrm{MSA}(\mathrm{LN}\left(Z_{l-1}^{\left(i\right)}\right))\right)+Z_{l-1}^{\left(i\right)},\\ Z_{l}^{\left(i\right)} & =\mathrm{DropPath}\left(\mathrm{FFN}(\mathrm{LN}\left({\hat{Z}}_{l}^{\left(i\right)}\right))\right)+{\hat{Z}}_{l}^{\left(i\right)}, \end{array}$$

where W−MSA represents window-based multi-head self-attention, LN is layer normalization, and FFN is the feedforward network with GELU activation and DropPath [26]. For stage *i*, the first input is given by $Z_{0}^{\left(i\right)}\equiv Z_{\mathrm{start}}^{i}$. The output of the final block defines the stage-level visual representation $V_{i}\in R^{N_{i}\times d_{i}}$, where $N_{i}=H_{i}\;\cdot \;W_{i}$ and $d_{i}\in \left\{d,\;2d,\;4d,\;8d\right\}$ for i=1,…,4.

The spatial resolution is progressively halved, and the channel capacity is doubled via patch-merging layers. This hierarchical design allows the model to maintain a large receptive field for global context while preserving high-resolution feature maps for small-object localization. To bridge the gap between fine-grained textures and high-level semantics, we define hierarchical feature flows. The SAAM (see Section 3.4) integrates the linguistic context into the visual stream through a residual connection prior to downsampling. The inter-stage information flow is

(3) $$\left\{\begin{array}{c} V_{i}=T_{i}\left(Z_{\mathrm{start}}^{i}\right),\\ C_{i}=\mathrm{SAAM}\left(V_{i},\;L\right),\\ {\hat{V}}_{i}=F_{\mathrm{fuse}}\left(V_{i},\;C_{i}\right),\\ Z_{\mathrm{start}}^{i+1}=M\left({\hat{V}}_{i}\right),\;i=1,2,3, \end{array}\right.$$

where $T_{i}\left(\cdot \right)$ is the stacked $L_{i}$ Swin Transformer blocks of stage *i* and $F_{\mathrm{fuse}}\left(\cdot \right)$ is the gated cross-modal residual fusion function defined in Equation (10). For the final stage (i=4), no patch merging M(·) is applied.

### 3.3. Language Encoder

The language encoder utilizes a pretrained BERT base-uncased model to extract multi-level semantic representations from the input query. A question *Q* is first tokenized using the WordPiece tokenization, generating a sequence $T=[t_{\mathrm{cls}},t_{1},t_{2},\ldots ,t_{m}]$, where *m* denotes the sequence length. These tokens are converted to embeddings and augmented with positional encodings:

(4) $$H_{0}^{L}=E_{\mathrm{word}}\left(T\right)+E_{\mathrm{pos}}^{L}+E_{\mathrm{seg}}^{L},$$

where $E_{\mathrm{word}}\left(\cdot \right)$ is the token embedding mapping and $E_{\mathrm{pos}}^{L}$ and $E_{\mathrm{seg}}^{L}$ are the BERT positional and segment encodings, respectively. The resulting embeddings traverse 12 stacked Transformer encoder layers:

(5) $$\begin{array}{cc} {\tilde{H}}_{l}^{L} & =\mathrm{LN}\left(\mathrm{MHSA}\left(H_{l-1}^{L}\right)+H_{l-1}^{L}\right),\\ H_{l}^{L} & =\mathrm{LN}\left(\mathrm{FFN}\left({\tilde{H}}_{l}^{L}\right)+{\tilde{H}}_{l}^{L}\right), \end{array}$$

where l=1,…,12. MHSA denotes multi-head self-attention. The output from the last layer $H_{12}^{L}\in R^{B\times N_{l}\times D}$ (D=768) is rearranged to a channel-first format as $L={\left(H_{12}^{L}\right)}^{\;⊤}\in R^{B\times D\times N_{l}}$. The channel-first convention aligns L with the pointwise convolution operations adopted in the SAAM.

### 3.4. Spatial Attention Aggregation Module

Cross-modal fusion methods for RS-VQA conventionally derive a single global language vector and apply it uniformly across all spatial positions [6,7]. Given that semantic relevance is determined differently across spatial locations, the spatially invariant weighting is inappropriate for RS imagery. Applying an identical fusion coefficient across such heterogeneous regions conflates semantically distinct content and degrades localization of small, query-relevant objects. A further consequence of late-stage fusion is that patch-merging operations progressively discard high-frequency spatial detail before any linguistic conditioning is applied, rendering the loss of question-relevant fine-grained features irreversible. To address both limitations, the SAAM computes per-position fusion weights dynamically conditioned on the input question and applies them at every backbone stage, ensuring that linguistic signals modulate visual feature extraction continuously throughout the spatial hierarchy.

#### 3.4.1. Cross-Modal Spatial Attention

The fusion weights for each position are influenced by the determination of spatial correspondences between the tokens of the question and the regions of the image. A multi-head query-key-value formulation is adopted, wherein each attention head captures a distinct semantic aspect of the query-object category, cardinality, or spatial relation and localizes it to the corresponding image region. Instance normalization is applied to the query branch in preference to batch normalization because the question length and vocabulary vary substantially across samples; instance statistics are computed independently per sample, producing stable attention score distributions regardless of sequence length variability, whereas batch statistics would be distorted under such conditions.

Given visual features $V_{i}$ from stage *i* and linguistic features L, the SAAM first projects the visual features through a gated projection layer:

(6) $$V_{i}^{\prime }=\delta \left({\mathrm{Conv}}_{1\times 1}\left(V_{i}\right)\right),$$

where ${\mathrm{Conv}}_{1\times 1}$ represents a pointwise convolution applied along the channel dimension and δ(·) is a nonlinear activation block consisting of a GELU activation function and dropout regularization.

Subsequently, the SAAM derives language-guided features through multi-head spatial cross-attention. The cross-modal interaction is formulated as a multi-head query-key-value mechanism:

(7) $$\begin{array}{cc} Q_{i} & =\mathrm{IN}(P_{Q}\left(V_{i}^{\prime }\right)),\\ K_{i} & =P_{K}\left(L\right)⊙M_{L},\\ U_{i} & =P_{U}\left(L\right)⊙M_{L}, \end{array}$$

where $P_{Q},P_{K}$, and $P_{U}$ are pointwise projections with 1×1 convolutions for queries, keys, and values, IN(·) is the instance normalization applied to the query branch, and $M_{L}\in {\left\{0,1\right\}}^{1\times N_{l}}$ is the binary attention mask that zeroes out padded positions before the attention computation. The multi-head attention scores and attended output are computed as follows:

(8) $$\begin{array}{cc} A_{i} & =\mathrm{Softmax}\left(\frac{Q_{i}^{⊤}K_{i}}{\sqrt{d_{k}}}+{\tilde{M}}_{L}\right),\\ L_{\mathrm{att}}^{i} & =\mathrm{IN}\left({\mathrm{Conv}}_{1\times 1}\left(U_{i}\;A_{i}^{⊤}\right)\right), \end{array}$$

where $d_{k}=d_{i}/n_{h}$ is the per-head key dimension with $n_{h}$ attention heads, ${\tilde{M}}_{L}$ denotes the additive attention mask obtained from the binary mask $M_{L}$, and ${\mathrm{Conv}}_{1\times 1}$ followed by instance normalization (IN) constitutes the output projection that maps the attended features back to $R^{d_{i}}$.

#### 3.4.2. Spatially Adaptive Gating Mechanism

The spatial attention aggregation module (SAAM) is designed to perform dynamic, language-conditioned gating across the spatial domain. Standard fusion methods often apply a global weight to the entire image, which fails in RS scenes where the relevance of different regions, e.g., a target building versus a background forest, varies significantly based on the question. The SAAM computes the per-pixel fusion coefficients for granular interaction between modalities. Given the question-guided features $L_{\mathrm{att}}^{i}$, a lightweight two-layer convolution path is calculated for the position-specific weight map $\omega_{i}=\sigma \left(\mathrm{MLP}\left(L_{\mathrm{att}}^{i}\right)\right),\omega_{i}\in {\left[0,1\right]}^{d_{i}\times N_{i}}$, and σ is the sigmoid function. The final gated fused feature $C_{i}$ for stage *i* is

(9) $$C_{i}=F_{P}\left(\omega_{i}⊙V_{i}^{\prime }+\left(1-\omega_{i}\right)⊙L_{\mathrm{att}}^{i}\right),$$

where ⊙ is the Hadamard product and $F_{P}\left(\cdot \right)$ represents a feature projection block composed of a 1×1 convolution, GELU activation, and dropout. Since $\omega_{i}$ is spatially resolved, large homogeneous regions that are visually unambiguous (e.g., farmland or water bodies) are assigned greater visual weights. Conversely, cluttered, small-object, and confusion-prone regions receive stronger question-guided linguistic support. This gating mechanism reduces extraneous noise and boosts question-specific targets.

A residual connection captures the initial visual representations. Meanwhile, $C_{i}$ is independently passed through a stage-specific layer normalization and reshaped into a spatial feature map for the PFCM:

(10) $$\begin{array}{cc} {\hat{V}}_{i} & =V_{i}+\tanh\;\left(\mathrm{MLP}(C_{i})\right)⊙C_{i},\\ {\bar{C}}_{i} & =R_{N_{i}\to H_{i}\times W_{i}}\left(\mathrm{LN}\left(C_{i}\right)\right), \end{array}$$

where R denotes the spatial reshaping operator. The residual output ${\hat{V}}_{i}$ is forwarded to the patch-merging layer for downsampling.

### 3.5. Progressive Feature Fusion and Classification Module

The PFCM tackles the scale mismatch problem by incrementally refining the multi-scale cross-modal pyramid (${\left\{{\bar{C}}_{i}\right\}}_{i=1}^{4}$) created by each of the four SAAM instances. Specifically, low-level features $\left({\bar{C}}_{1},{\bar{C}}_{2}\right)$ preserve fine-grained spatial details, whereas high-level features $\left({\bar{C}}_{3},{\bar{C}}_{4}\right)$ encode abstract semantic representations. Direct concatenation of such heterogeneous scales often leads to information dilution. The PFCM therefore employs a two-stage channel reduction strategy to retain inter-scale correlations while controlling the computational cost.

#### 3.5.1. Channel Projection and Spatial Alignment

Given normalized multi-scale features ${\bar{C}}_{i}\in R^{d_{i}\times H_{i}\times W_{i}}$, the PFCM first projects all features to a unified channel dimension *h*, and higher-resolution maps are resampled to match the smallest spatial extent via bilinear interpolation:

(11) $$F_{i}=I_{\mathrm{align}}\left({\mathrm{Conv}}_{1\times 1}\left({\bar{C}}_{i}\right)\right)$$

where $I_{\mathrm{align}}\left(\cdot \right)$ is the spatial alignment operation via bilinear interpolation.

#### 3.5.2. Feature Concatenation and Progressive Fusion

The spatially aligned features are stacked over the channels and reduced by two subsequent convolutional layers. Relative to a direct one-step projection, the progressive channel reduction strategy has greater efficiency in preserving inter-scale correlations:

(12) $$F_{\mathrm{cat}}=\left[F_{1};\;F_{2};\;F_{3};\;F_{4}\right]\in R^{4h\times H_{4}\times W_{4}},$$

(13) $$\begin{array}{cc} F_{\mathrm{mid}} & =\delta \left(B\left({\mathrm{Conv}}_{3\times 3}\left(F_{\mathrm{cat}}\right)\right)\right)\in R^{2h\times H_{4}\times W_{4}},\\ F_{\mathrm{fused}} & =\delta \left(B\left({\mathrm{Conv}}_{3\times 3}\left(F_{\mathrm{mid}}\right)\right)\right)\in R^{h\times H_{4}\times W_{4}}, \end{array}$$

where δ(·) and B(·) are the ReLU activation function and batch normalization, respectively.

### 3.6. Answer Prediction and Training Objective

#### 3.6.1. Answer Prediction

The fused feature map $F_{\mathrm{fused}}$ is first spatially aggregated by global average pooling (GAP) and then classified through a two-layer perceptron:

(14) $$y=\mathrm{MLP}(\mathrm{GAP}\left(F_{\mathrm{fused}}\right)),$$

where $y\in R^{K}$ is the predicted scores over *K* categories.

#### 3.6.2. Training Loss

Given predicted logits $y_{n}$ and the ground-truth label index ${\hat{k}}_{n}$ for the *n*th sample, the model is optimized with cross-entropy loss:

(15) $$L=-\frac{1}{N}\sum_{n=1}^{N}\log\frac{\exp(y_{n}^{\left({\hat{k}}_{n}\right)})}{\sum_{j=1}^{K}\exp\left(y_{n}^{\left(j\right)}\right)},$$

where *N* is the batch size.

## 4. Results

### 4.1. Datasets and Evaluation Metrics

#### 4.1.1. Datasets

We evaluated the PMA-VQA on the three benchmark datasets of RS-VQA LR, RS-VQA HR, and HRVQA:

- **RS-VQA LR** [6] is based on low-resolution imagery from the Sentinel-2 satellites. It contains 772 images paired with 77,232 question-answer sets. The queries cover categories such as counting, presence, comparison, and rural and urban classification.
- **RS-VQA HR** [6] is composed of 10,659 high-resolution aerial orthoimages (512×512) and approximately 1.06 million question-answer pairs. The types of questions include counting objects, presence identification, comparison, and area identification.
- **HRVQA** [27] comprises 53,512 high-resolution aerial orthoimages (1024×1024 pixels) at an 8 cm ground sampling distance acquired over four Dutch cities, paired with 1,070,240 question-answer pairs across 10 question types covering fine-grained attribute categories, including color, shape, location, transportation, and sports.

#### 4.1.2. Evaluation Metrics

Following previous RS-VQA studies, we utilized the overall accuracy (OA) and average accuracy (AA) as the evaluation metrics. We also offer per-question-type model reasoning breakdowns to measure the model reasoning ability for each question type.

### 4.2. Implementation Details

#### 4.2.1. Network Configuration

The proposed framework adopts a dual-stream architecture. For the visual stream, a Swin Transformer Base backbone is employed as the image encoder, comprising four hierarchical stages with channel dimensions of {128,256,512,1024} (i.e., {d,2d,4d,8d}, where d=128) with attention head counts of {4,8,16,32}, yielding a constant per-head key dimension of $d_{k}\;=\;32$ across all stages. For the language stream, BERT is adopted as the text encoder with a maximum sequence length of m=30; only the first 10 Transformer layers are fine-tuned to balance domain adaptation and computational efficiency, with the remaining layers kept frozen. The visual encoder is initialized with weights pretrained on ImageNet-22K, and the language encoder adopts BERT base-uncased pretrained weights.

#### 4.2.2. Training Configuration

All models were trained using the AdamW optimizer with an initial learning rate of $5\times 10^{-5}$ and a weight decay of $1\times 10^{-2}$, with the learning rate decayed via a polynomial schedule to ensure stable convergence. A dropout rate of 0.1 was applied in the classification head for regularization. Training was conducted on four NVIDIA RTX 3090 GPUs (NVIDIA Corporation, Santa Clara, CA, USA) under a distributed data parallel (DDP) strategy, with a per-GPU batch size of 16 (total batch size of 64). The software environment included PyTorch 1.7.1 and CUDA 11.0. The model was fine-tuned for 10 epochs on RS-VQA LR and 5 epochs on RS-VQA HR, following the official dataset splits throughout. To assess reproducibility, two additional runs with independent random seeds were performed. The per-run results and descriptive statistics are reported in Appendix B (Table A2), confirming stable convergence with an OA standard deviation below 0.05% across all benchmarks. Following the official city-level partition, the model was trained on Utrecht and Rotterdam and evaluated on the geographically disjoint Enschede split for HRVQA. As the Amsterdam test split used in the original HRVQA paper [27] is not publicly available, the Enschede validation split served as the evaluation set for all compared methods, ensuring protocol consistency across all reported results.

### 4.3. Compared Methods

To evaluate our proposed approach, a series of experiments was performed on two popular datasets, RS-VQA HR and RS-VQA LR, to compare our proposed method with existing ones:

- RS-VQA [6]: A pioneering baseline leverages dot-product fusion between visual and textual features to predict the answers.
- EasyToHard [13]: A progressive learning approach with the self-paced curriculum learning (SPCL) strategy. It learns from easy to hard questions for multi-level visual feature learning.
- BiModa [28]: A vision–language Transformer encoder-decoder with both self-attention and co-attention blocks for intra-modal modeling of features and cross-modal units of dependencies to realize efficient feature fusion and prediction.
- SHRNet [29]: A hash-based spatial multi-scale visual learning model which increases position awareness for image processing operations such as extraction and fusion.
- SAMFPN [7]: A scale-aware multi-level feature pyramid network that captures multi-scale contextual information. It aggregates features across different scales via a coordinate attention mechanism.
- HFIF [14]: A hierarchical feature integration and fusion framework. It employs multi-granularity correlation learning and dilated convolutions to bridge the semantic gap between global and local specifics.
- RSGPT [16]: An RS generation pretrained large model fine-tuned with InstructBLIP. It aligns RS image features with large language models through data-efficient training, thereby advancing performance in VQA and associated tasks.
- SkyEyeGPT [17]: A unified multimodal large language model specifically designed for RS vision–language understanding. It utilizes a simplified architecture consisting of a frozen visual encoder, a linear alignment layer to bridge the modality gap, and an LLaMA 2-based decoder.
- GFTransformer [27]: The baseline model proposed alongside the HRVQA dataset, serving as the external reference point for our generalization analysis in Section 4.5. It combines gated positional co-attention units with a mutual fusion module, using ResNet-152 grid features for visual representation and GloVe embeddings followed by an LSTM encoder for language encoding.

### 4.4. Main Results

To verify the efficacy of the proposed PMA-VQA framework, we conducted comparative experiments on RS-VQA LR and RS-VQA HR against existing state-of-the-art methods. A supplementary evaluation on HRVQA examining city-level robustness and question-type diversity is presented in Section 4.5. For the RS-VQA LR and HR evaluation, we categorized the baseline methods into three groups: (1) pioneering shallow fusion models (RS-VQA), (2) specialized hierarchical and multi-scale attention networks, including EasyToHard [13], Bi-Modal [28], SHRNet [29], SAMFPN [7], and HFIF [14], and (3) emerging multimodal large language models (MLLMs) tailored to remote sensing, including RSGPT [16] and SkyEyeGPT [17].

To ensure a fair comparison, the results for all compared methods were directly cited from the respective original publications, conforming to the evaluation protocols and data partitions established in prior RS-VQA work. The results reproduced in our experiments are marked with ^†^ in the comparison tables; all other results are cited from the original publications.

#### 4.4.1. Results on the RS-VQA LR Dataset

As summarized in Table 2, PMA-VQA achieved state-of-the-art performance across the primary metrics on the RS-VQA LR dataset. Our model outperformed both the traditional CNN-based models and modern MLLM-based models, with an OA of 87.38% and an AA of 89.25%. Compared with specialized multi-scale models like the SAMFPN and HFIF, PMA-VQA outperformed them in terms of OA by 2.62% and 0.68%, respectively. HFIF leverages multi-granularity correlation and dilated convolutions to bridge the semantic gap, and PMA-VQA fundamentally advances this by introducing a progressive multi-scale fusion strategy. Our model showed considerable superiority in the “Count” category with an accuracy of 76.04%, which was 1.43% better than HFIF and 2.17% better than SHRNet. Such improvement validates that our framework more effectively preserves the fine-grained spatial details necessary for enumerating small, densely packed geographical entities on low-resolution imagery.

Additionally, PMA-VQA showed more precision than the MLLMs (SkyEyeGPT and RSGPT) when it came to the task of discrimination for classification of “Rural or Urban”. While MLLMs are good at generating unbounded open-ended text, our model aligned multi-level language guidance with visual components, allowing more solid grounding than MLLMs for remote sensing scene understanding.

#### 4.4.2. Results on the RS-VQA HR Dataset

Table 3 and Table 4 present the quantitative results of PMA-VQA and comparative methods on the RS-VQA HR dataset. As demonstrated in Table 3, PMA-VQA attained an 85.59% AA and 85.90% OA on Test Set 1, consistently outperforming all evaluated baselines. Our approach obtained the highest results for the “Count” (70.90%), “Comparison” (92.31%), and “Area” (86.90%) categories. These three types of queries demand precise numerical reasoning, fine-grained relational reasoning, and region-level semantic interpretation, respectively. For the “Presence” category, HFIF yielded a marginally higher score of 92.58% versus 92.27% for our model. This result could be due to the dilated convolutional architecture used by HFIF, which efficiently increases the spatial reach of feature extraction while maintaining the resolution. This might offer a minor edge in binary presence detection, where comprehension of the entire scene is required. In contrast, the PMA-VQA model’s spatially adaptive gating mechanism attempts to adjust attention at a more granular level, providing increased benefits in tasks requiring the discrimination of spatially close or semantically related entities.

Table 4 further confirms the reliability of PMA-VQA on Test Set 2, which spanned a different geographical area. PMA-VQA achieved the highest accuracy for the two categories “Comparison” (90.36%) and “Area” (80.94%), proving that progressive multi-scale fusion is effective for relational and region-level reasoning. With regard to the “Count” category, PMA-VQA (62.78%) was surpassed by SHRNet (63.42%), HFIF (63.29%), and Bi-Modal (63.06%). This discrepancy outlines an underlying trade-off present in hierarchical cross-modal fusion architectures: optimization of global relational reasoning and spatial-semantic grounding may unintentionally undercut dense, instance-level quantification. RSGPT obtained the top “Presence” accuracy result of 89.87%. Regardless of these differences between categories, PMA-VQA showed the best overall performance across both evaluation splits. By leveraging multi-level semantic guidance to progressive feature fusion, our PMA-VQA addresses scale ambiguity for high-resolution cases.

### 4.5. Generalization Analysis on HRVQA

Table 5 reports the results on the HRVQA Enschede validation split under the official city-level partition, with models trained on Utrecht and Rotterdam and evaluated on the geographically disjoint Enschede set. The full PMA-VQA achieved an OA of 88.36% and an AA of 87.26%, consistently outperforming the reproduced GFTransformer baseline, with an OA of 80.57% and AA of 80.67%, as well as all ablated variants.

The fully configured model achieved the highest accuracy for the most challenging visual discrimination categories, namely “Shape” at 94.26%, “Y/N” at 93.34%, “Numbers” at 77.13%, and “Scene” at 79.34%. The “Scene” category benefited from the complete model in a manner consistent with the region-level semantic coherence effect provided by progressive multi-scale fusion seen in the RS-VQA HR “Area” results.

The most informative ablation signal comes from the “Location” category. The full PMA-VQA model at 78.30% recovered 10.60 percentage points with respect to w/o SAAM’s 67.70%, which shows that per-position gating captures the topological relations of the landmarks and the location-type queries. Furthermore, the w/o PFCM variant even underperformed relative to the dual-ablation baseline for the “Location” category, with 73.29% against 78.68%, suggesting that deploying the SAAM without multi-scale pyramid integration biases the model toward local discriminative features at the expense of broader spatial context. The collaborative effect of the SAAM and PFCM in spatial relational reasoning aligns with the joint-module results on RS-VQA HR for the “Comparison” and “Area” categories.

GFTransformer led for the “Areas” category at 99.75% against 88.93% for PMA-VQA. “Area” queries in HRVQA predominantly involve binary classification of large, spectrally homogeneous land cover regions, a setting that favors the global grid features of ResNet-152 over hierarchical window-attention representations, and fine-grained spatial cues from the SAAM and PFCM offer limited complementary value under these conditions.

Overall, PMA-VQA achieved the best OA and AA on the geographically unseen Enschede split, and the relative contributions of the SAAM and PFCM across question types were directionally consistent with the RS-VQA LR and HR ablation findings, supporting the generalization of the proposed framework beyond the RS-VQA dataset family.

### 4.6. Ablation Studies

Ablation studies were conducted on the RS-VQA LR and RS-VQA HR datasets to analyze the contributions of the SAAM and PFCM modules, as shown in Table 6. To ensure fairness and rigor in the experimental comparisons, we adopted a module replacement strategy rather than a direct ablation setting. Specifically, in the SAAM variant, the proposed SAAM module is replaced with a standard cross-attention fusion mechanism. For the PFCM comparison, we substituted the proposed module with a basic MLP classifier.

As seen in Table 6, the baseline model showed considerable improvement by only adding the PFCM module. In particular, the AA for the RS-VQA LR dataset increased from 83.06% to 87.12%, or an increase of four percentage points, while the OA increased from 83.49% to 85.87%. The PFCM uses a hierarchical module for the aggregation of multi-scale features. That improves the alignment of visual-semantic information across diverse layers and accurately discriminates target objects with enhanced precision. The variant with only the SAAM module also exceeded the baseline, recording an AA of 87.75% on the LR dataset. Our framework utilizes language-based spatially adaptive gating to control visual attention to task-oriented areas, which translates to an increased alignment of visual understanding and implicit question intent.

When both modules operated jointly, the complete PMA-VQA architecture yielded the highest scores across all evaluation benchmarks. On the LR dataset, the dual-module configuration reached an AA of 89.25% and an OA of 87.38%, surpassing the baseline variant—where standard cross-attention replaced the SAAM and an MLP classifier substituted the PFCM—by 6.19 and 3.89 percentage points in terms of the AA and OA, respectively. It bears emphasis that this replacement baseline itself constitutes a competitive cross-modal attention architecture. Consequently, the consistent accuracy gains substantiate that spatially adaptive gating and progressive multi-scale fusion each deliver targeted functional improvements rather than merely introducing additional model capacity. On the RS-VQA HR benchmarks, the complete model recorded an OA of 85.90% on Test Set 1 and 81.55% on Test Set 2, confirming that the complementary mechanisms embedded in the SAAM and PFCM maintained their effectiveness under varying image resolutions and across distinct geographical domains. The directional consistency of these module contributions was further corroborated by the HRVQA ablation analysis in Table 5, where the relative ordering of the four configurations mirrors that observed here under a substantially different question-type distribution.

#### 4.6.1. Fine-Grained Ablation of SAAM Components

To further investigate the internal mechanisms of the SAAM, we conducted a component-level ablation study on the RS-VQA LR dataset. As summarized in Table 7, three constituent components were progressively incorporated: (1) the cross-modal spatial attention mechanism (Equation (8)), (2) the spatially adaptive gating mechanism (Equation (9)), and (3) the residual gating connection (Equation (10)).

The baseline using conventional cross-attention without any additional SAAM components achieved an OA of 85.87% and an AA of 87.12%. Adding the residual gating connection alone (row 2) increased the OA by 0.60 pp to 86.47%, and the largest per-category increase was found for the “Count” category with 1.96 pp. The tanh-bounded residual bypass is designed to preserve the high-frequency spatial components in $V_{i}$ that are likely to be lost due to cross-modal attention, which is critical in tasks of instance-level enumeration, where detailed boundary information determines whether adjacent objects are identified as separate targets.

Improved performance, where the OA increased by 1.10 pp to 86.97% and the AA increased by 1.83 pp to 88.95%, can be seen when a spatially adaptive gate was introduced without a residual connection. More notable gains can be seen in “Rural or Urban” with +4.00 pp and “Count” with +2.70 pp. In discrimination of land use at the scene level, the position-specific gating weights allowed the model to selectively amplify the linguistic context in areas where urban and rural spectra intersect, while maintaining the visual representation in areas that are spectrally uniform. In counting tasks, the use of per-position gating weights aligns with the spatial selectivity hypothesis of the gate design as it focuses on the tightly clustered observations and reduces the impact of unwanted supportive background elements.

The complete SAAM configuration, including all components in row 4, obtained an OA of 87.38% and an AA of 89.25%, demonstrating the highest performance across all question types. The gradual advancement from row 1 to row 4 indicates that these three mechanisms work together cooperatively rather than redundantly. The cross-modal spatial attention focuses on building query-to-region relationships; the spatially adaptable gate addresses per-position modality weighting, and the residual gating connection stabilizes cross-modal feature learning by controlling the degree of modulation during optimization.

#### 4.6.2. Progressive Multi-Scale Fusion Analysis

Table 8 details the ablation study concerning the step-wise addition of cross-modal feature maps to the PFCM on the RS-VQA LR dataset. Starting from the deepest representation ${\bar{C}}_{4}$ alone (OA = 86.38%), each additional scale level brought a consistent accuracy gain; the OA increased to 86.67% upon incorporating ${\bar{C}}_{3}$ and further to 86.99% with ${\bar{C}}_{2}$, and it attained 87.38% when all four levels were fused (${\bar{C}}_{1:4}$), confirming that each scale level contributed non-redundant information.

A category-wise analysis revealed distinct scale preferences across the question types. The “Rural or Urban” classification achieved its peak accuracy of 99.00% using ${\bar{C}}_{4}$ alone and declined marginally by 2.00 pp as finer-grained features were incorporated, indicating that scene-level land use discrimination was predominantly determined by high-level semantic context, for which spatially detailed representations offered limited complementary information. In contrast, the “Count” category exhibited the largest cumulative gain of 2.27 pp, rising from 73.77% to 76.04%, as accurate object enumeration requires both fine-grained boundary cues from early stages to delineate individual instances and high-level semantic discriminability from late stages to suppress visually similar distractors. The “Comparison” category’s accuracy improved steadily from 91.55% to 92.27%, consistent with the multi-scale spatial reasoning inherent in queries involving objects of varying sizes.

These results collectively demonstrate three properties of the progressive fusion design: (1) each scale level provides complementary, non-redundant information; (2) the marginal OA contribution of each additional level is consistently positive; and (3) the inherent trade-off between semantic abstraction and spatial precision across question types is effectively resolved through unified progressive fusion without requiring task-conditioned decoding heads.

### 4.7. Qualitative Analysis

#### 4.7.1. Attention Heat Map Visualization

**Figure 3:**
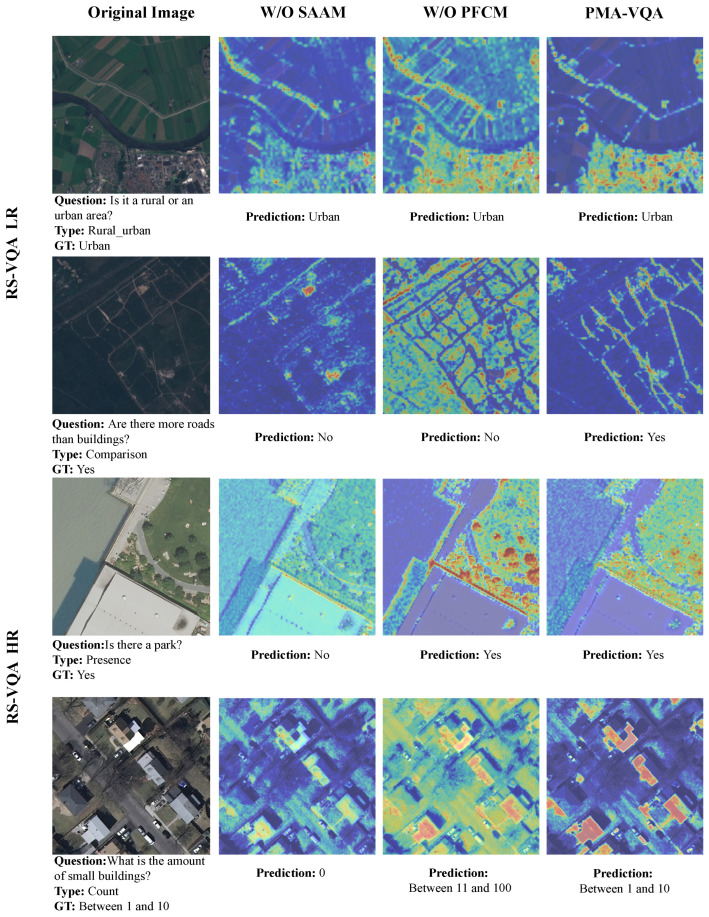
Grad-CAM visualizations [30] of PMA-VQA on the RS-VQA LR and RS-VQA HR datasets. From left to right in the columns are the remote sensing original images, the model without the spatial adaptive attention module (“w/o SAAM”), the model without the progressive feature fusion and classification module (“w/o PFCM”), and the complete PMA-VQA model.

Extensive visualization experiments were conducted using Grad-CAM attention heat maps [30]. As illustrated in Figure 3, a consistent observation emerged across both benchmarks: the proposed PMA-VQA exhibited notably more concentrated and precisely localized feature separation on semantically relevant entities. Conversely, the lack of individual modules resulted in high degrees of attention drift or diffuse saliency maps.

Regarding the low-resolution RS-VQA LR dataset, the first row reveals that although all variants eventually converged on the correct answer, the attention maps for the “w/o SAAM” and “w/o PFCM” configurations remained insufficiently focused on the target objects. This validates that the SAAM’s gating mechanism efficiently refines local positioning under linguistic guidance and the PFCM mitigates the loss of low-level spatial detail, resulting in better spatially aligned attention. The impact of module exclusion is presented in the second row, which shows how only the complete model was able to isolate the target cluster. In particular, the “w/o SAAM” variant showed bias toward the high contrast surrounding the distraction areas, e.g., neighboring buildings, while disregarding the road layout. Meanwhile, the “w/o PFCM” variant displayed localized excessive response and yielded erroneous building counts.

On the RS-VQA HR dataset, the third row shows that the “w/o PFCM” variant achieved the correct answer, but PMA-VQA maintained sharper focus on small-scale targets with better suppression of background distractors. The fourth row presents a critical failure case; the “w/o SAAM” model predicted “0”, owing to imprecise multimodal alignment, the “w/o PFCM” model predicted “10–100”, owing to inadequate scale-feature integration, while only the complete PMA-VQA correctly identified the count to be within the “1–10” range. These visualizations corroborate the indispensable roles of the SAAM in semantic alignment and the PFCM in scale discrimination and small-object quantification.

#### 4.7.2. Human Evaluation

To quantify the degree to which each model variant grounded its predictions in question-relevant spatial evidence, we conducted a structured human evaluation of the Grad-CAM attention heat maps.

Three annotators with remote sensing image interpretation experienced independently scored Grad-CAM heatmaps for 50 randomly sampled RS-VQA LR test instances under a blinded protocol. For each sample, the three model variants were presented in a randomized order, and annotators were not informed of the model identity until all scores were recorded. Each heat map was evaluated on three ordinal criteria (0–2): (1) the spatial relevance score (SRS), showing whether the high-activation region spatially coincided with the image area required to answer the question, where two is highly relevant, one is partially relevant, and zero is irrelevant; (2) answer justification consistency (AJC), showing whether the predicted answer was visually grounded in the highlighted region, where two is fully justified, one is partially justified, and zero is unjustified; and (3) context sensitivity (CS), showing whether the model assigned spatially differentiated saliency across semantically distinct regions rather than uniform activation, where two is clearly discriminative, one is ambiguous, and zero is indiscriminate.

Table 9 reports the mean scores for each model variant. PMA-VQA attained the highest scores for all three criteria. The most pronounced gain was observed for the AJC; the full model surpassed “w/o SAAM” by +0.46 points, confirming that per-position linguistic gating in the SAAM directly strengthened the consistency between the predicted answer and the spatially highlighted evidence. It also outperformed “w/o PFCM” by +0.34 points, indicating that progressive multi-scale fusion in the PFCM provided additional complementary grounding. An SRS advantage of +0.34 over “w/o PFCM” further demonstrates that progressive multi-scale fusion in the PFCM anchored the attention to question-relevant regions more reliably across scales, consistent with the per-module attribution in Appendix D, where the “w/o SAAM” variant (which retained the PFCM) yielded an SRS of 1.24 versus 1.16 for the “w/o PFCM” variant. The CS gains of +0.28 over “w/o PFCM” and +0.36 over “w/o SAAM” provide direct evidence that the joint operation of the SAAM and PFCM enabled spatially differentiated saliency, a property not captured by aggregate accuracy metrics. A complete case study with Top-2 Rate statistics and extended case analysis is provided in Appendix D.

### 4.8. Counting Performance Analysis

As shown in Table 3 and Table 4, PMA-VQA achieved the highest counting accuracy of 70.90% on HR Test Set 1 yet was marginally surpassed by SHRNet at 63.42% and HFIF at 63.29% on HR Test Set 2, where PMA-VQA scored 62.78%. To determine whether this gap reflects an architectural limitation or a dataset-specific effect, we conducted a targeted error analysis.

**Figure 4:**
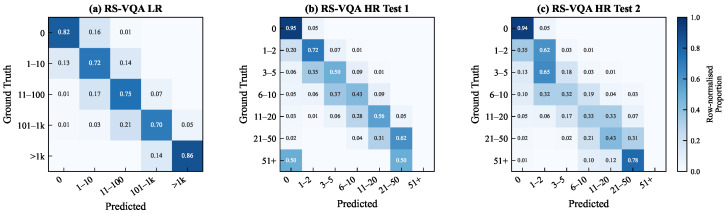
Row-normalized confusion matrices for counting predictions. (**a**) RS-VQA LR with ordinal bins. (**b**,**c**) RS-VQA HR with grouped integer ranges. Off-diagonal mass concentrated in adjacent bins, confirming that errors were predominantly near-misses.

**Figure A2:**
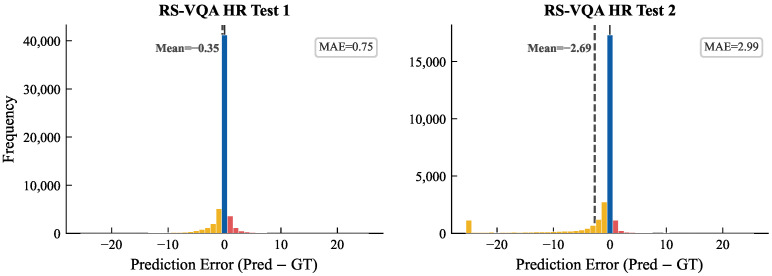
Signed prediction error distribution (Pred−GT) for counting questions on RS-VQA HR Test Set 1 and Test Set 2. Dashed lines indicate the respective mean errors: blue for exact match (error =0), red for overcounting (error >0), and amber for undercounting (error <0).

Decomposing predictions into exact matches, overcounts, and undercounts revealed a consistent *conservative undercounting* tendency, which intensified on HR Test Set 2, where the mean signed error shifted from −0.35 on Test Set 1 to −2.69. The row-normalized confusion matrices in Figure 4 confirm that errors were highly localized; 98.9%, 85.9%, and 76.8% of the mispredictions fell within ±1 ordinal bin of the ground truth on LR and HR Test Set 1 and HR Test Set 2, respectively, indicating near-miss boundary uncertainty rather than catastrophic failure. The signed error histograms (Figure A2 in Appendix C) further quantify this shift. The mean signed error moved from −0.35 (MAE = 0.75) on Test Set 1 to −2.69 (MAE = 2.99) on Test Set 2, with both distributions sharply concentrated around zero, confirming that large-magnitude errors were rare.

**Figure 5:**
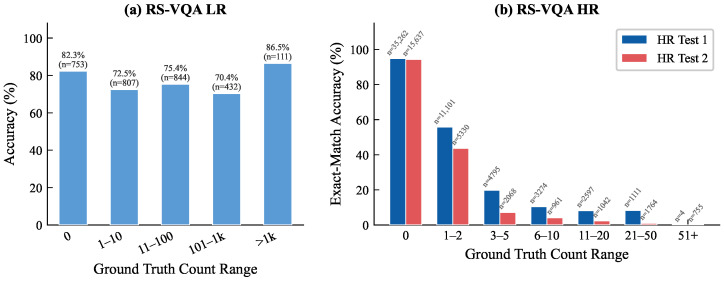
Exact match accuracy stratified by ground-truth count range. (**a**) RS-VQA LR with ordinal bins. (**b**) RS-VQA HR with grouped integer ranges. Sample sizes are annotated per bar.

The exact match accuracy as a function of the ground-truth count range, shown in Figure 5, revealed a monotonic decline beyond the 1–2 range on both HR splits, dropping below 10% for counts exceeding 10. This pattern is attributable to a severe long-tailed label imbalance, exemplified by the 35,262 zero-count queries that dominated HR Test Set 1, rather than a structural architectural deficiency. The sharper degradation on Test Set 2 is consistent with the geo-spatial domain shift between the two evaluation partitions.

The counting performance gap on HR Test Set 2 was primarily driven by long-tailed count distributions and a cross-split domain shift. PMA-VQA attained the highest counting OA on two of three splits and exhibited the narrowest RelAcc fluctuation (0.12 pp) under adversarial conditions, confirming robust and well-calibrated counting behavior overall.

### 4.9. Language Bias Evaluation

We conducted the bias evaluation to confirm the performance improvements of PMA-VQA resulting from geospatial reasoning as opposed to language priors. Following the work of Chappuis et al. [11], we report two critical bias indicators, *adversarial testing* (AdTest) and the *relative accuracy* (RelAcc), to disentangle visual understanding from language shortcuts.

The AdTest established a lower performance bound. It preserved the semantic consistency of the queries but withheld informative remote sensing imagery from the model. The image was whited, blacked out, or noised (each pixel was independently sampled from a uniform distribution). High accuracy AdTest suggests that the model is hallucinating answers based on language statistics. RelAcc quantifies the effective contribution of visual features to the final prediction. This metric normalized the standard OA against the language prior captured by AdTest:

(16) $$RelAcc=\frac{OA-AdTest}{100-AdTest}\times 100,$$

where a higher value of RelAcc shows a stronger dependency on visual evidence.

Table 10 shows the bias evaluation results on the RS-VQA LR and RS-VQA HR (Test 1 and Test 2) datasets. The full PMA-VQA consistently attained the highest OA on every benchmark while simultaneously maintaining the leading RelAcc. On the RS-VQA LR dataset, PMA-VQA attained the highest RelAcc under all three strategies, i.e., black (75.15%), white (75.37%), and noise (74.94%). It shows that most of the improvements in its accuracy came from visual evidence.

On the more challenging HR test sets, PMA-VQA maintained the best OA while simultaneously achieving the highest RelAcc across all corruption types. On Test Set 1, it attained the lead RelAcc under the black (64.07%), white (62.91%), and noise (62.71%) strategies, surpassing both ablated variants uniformly and confirming that the accuracy advantage stemmed from strengthened visual grounding rather than linguistic shortcuts. On Test Set 2, PMA-VQA reported the best results for RelAcc and showed that the complete model transferred its debiasing capacity more effectively to unseen geographical domains than either ablated variant.

The ablation patterns reveal that the two modules address distinct facets of language bias. Excluding the PFCM yielded a competitive RelAcc of 71.91% under black corruption on the LR benchmark and 73.01% under noise. Although both values were competitive, they remained over three percentage points below the full PMA-VQA (75.15% and 74.94%, respectively), confirming that progressive multi-scale fusion improves visual grounding for all families of corruption. On HR Test 1, the full PMA-VQA outperformed this PFCM-ablated variant in all three corruption scenarios (e.g., black: 64.07% vs. 63.27%), which shows that progressive fusion contributes positively to visual dependency even when the input signal is entirely removed. On HR Test 1, both the “w/o SAAM” and “w/o PFCM” variants exhibited uniformly lower RelAcc than the full model. With respect to both unified mechanisms, PMA-VQA integrated these complementary abilities and offered the most stable and consistent debiasing behavior over all the perturbation settings and resolution scales.

A finer-grained category-level analysis, reported in Table A1 of Appendix A, offers additional insight. The most substantial debiasing improvements were observed for “Rural or Urban” queries on the LR dataset, where PMA-VQA surpassed the SAAM- and PFCM-ablated variants by 9.09 and 6.82 percentage points, respectively. For “Comparison” queries, PMA-VQA achieved RelAcc gains of up to 6.00 percentage points over the ablated variants. The “Counting” category warranted separate examination. In the case of the LR dataset, PMA-VQA performed best in counting RelAcc under the three corruption conditions (64.23% black, 64.20% white, and 64.32% noise), better than both the PFCM-ablated variant (62.58%, 62.53%, and 62.24%, respectively) and SAAM-ablated variant (59.02%, 59.87%, and 61.13%, respectively). Notably, PMA-VQA maintained a RelAcc fluctuation of only 0.12 percentage points, compared with 0.34 for the PFCM-ablated variant and 2.11 for the SAAM-ablated variant, representing the most stable counting behavior among all evaluated configurations.

The bias evaluation yielded two principal insights. To begin with, the accuracy stated for PMA-VQA in Section 4.4 could be related to improved visual reasoning. The model simultaneously achieved the best OA and the leading RelAcc under all three corruption settings on both the LR and HR benchmarks. This confirms that the improvements in performance were based on real visual evidence. Then, the ablation study revealed an additional debiasing mechanism; the SAAM most significantly reduced bias when the visual signal was completely absent (as indicated by the significant black RelAcc drop when it was removed on LR), while the PFCM maintained visual grounding even when some corruption was present (as shown by the noise RelAcc drop when it was removed). Their joint deployment yielded the most stable and well-balanced debiasing performance across corruption types, resolution scales, and geographical domains. The results substantiate the progressive multi-scale design as a principled strategy for reducing linguistic shortcuts in remote sensing visual question answering.

### 4.10. Computational Cost Analysis

The complexity of four different PMA-VQA configurations is reported in Table 11. Relative to the baseline without the SAAM and PFCM, the full PMA-VQA model incurred a parameter increase of 9.7% (from 95.42 M to 104.64 M) and a FLOPs increase of 7.1% (from 47.15 G to 50.50 G). Importantly, there was only a 2.3% increase in GPU memory (from 1.034 to 1.058), suggesting that the new modules had minimal runtime overhead. The relatively small increase in memory usage compared with the increase in parameters was due to the efficiency of the design of the SAAM’s adaptive gating and the convolutional channel reduction of PFCMs, which improve parameters by using specific inductive biases instead of general capacity increasing. These two modules contributed 5.58 M and 3.65 M additional parameters, respectively, each with a clear functional role; the SAAM improved spatial selectivity via per-stage cross-attention projections, while the PFCM strengthened multi-scale semantic fusion. The resulting OA gains of 3.89 pp on RS-VQA LR and consistent improvements across both HR splits demonstrate that accuracy improvements stemmed from architectural design rather than parameter inflation alone.

## 5. Discussion

The inherent multi-scale object variability and spatial heterogeneity within remote sensing images present severe impediments to fine-detail geospatial reasoning. To address these challenges, we presented PMA-VQA, a progressive multi-scale feature fusion approach with spatially adaptive attention, which embeds linguistic guidance directly into hierarchical visual encoding stages rather than relying on late-stage or coarse-grained cross-modal integration. Experiments on RS-VQA LR and RS-VQA HR show that this stage-wise, language-conditioned alignment considerably improved scene comprehension accuracy. An especially interesting result is that PMA-VQA improved the most on the question types that involved detailed spatial discrimination and reasoning about objects at multiple scales, especially comparison and area estimation queries. This is consistent with the assumed hierarchical aggregation ability of the architecture. HFIF [14] and the SAMFPN [7] have shown that interacting features at multiple granularities sharpen cross-modal reasoning over aerial scenes. Our results reinforce that conclusion while further demonstrating the value of spatially adaptive fusion. The analysis of generalization on HRVQA further supports these findings, as the SAAM showed the biggest improvements on spatially discriminative categories like “Location” at +10.60 pp, while PFCM multi-scale relational reasoning was more beneficial, further confirming that the improvements noted were indeed functional contributions of the proposed modules.

We also revealed an additional complementary debiasing mechanism arising from the synergistic fusion of the two designed modules. Language bias analysis showed that the SAAM mainly reduced overreliance on textual statistical patterns in the complete absence of visual cues. This was validated by the significant decline in RelAcc under the black corruption test when the SAAM was removed. The PFCM further confirmed the dependency of the model on visual evidence in the case of partially degraded input remote sensing images. Its effectiveness was clearly reflected by the considerable performance gain under noise perturbation scenarios. The narrow variance in RelAcc across all three adversarial settings for the complete PMA-VQA—merely 0.43 percentage points on the LR benchmark—compared with considerably wider fluctuations for either ablated configuration substantiates that combining spatially adaptive gating with progressive scale integration yields the most balanced and resilient debiasing behavior. This finding extends beyond standard accuracy metrics and validates that the reported performance gains were due to visual grounding and not linguistic shortcuts, as quantified by RelAcc.

An unexpected observation was that for discrete object enumeration tasks, the complete hierarchical fusion architecture did not uniformly achieve the highest bias-adjusted accuracy. The appendix per-category analysis showed that some ablated variants achieved a better RelAcc in the counting category, such as the PFCM-ablated design for black and white conditions on the LR dataset and the SAAM-ablated variation for noise corruption. This result illustrates a trade-off of hierarchical cross-modal fusion. While progressive semantic injection excels at global relational reasoning, it can induce a “feature smoothing” effect in densely populated regions. However, in-depth evaluation validated that ablated models suffer from pronounced instability across varied perturbation groups relative to the proposed full network. PMA-VQA stood out as the most reliable method for quantitative analysis. When subjected to diverse adversarial perturbations, the deviation in its counting-related RelAcc was merely 0.12 percentage points. Such a high degree of stability was not observed in the PFCM-ablated or SAAM-ablated variations evaluated in this study. The contrasting insights from stability against different perturbations will guide future works, such as those on auxiliary density estimation or dynamic routing mechanisms.

Human evaluations combined with Grad-CAM visualizations operationalize spatial grounding quality as an externally validated construct, encompassing spatial activation co-localization, attention-answer justification consistency, and contextual spatial specificity, as detailed in Appendix D. We note that Grad-CAM, as a post hoc saliency method, provides an approximate rather than causal attribution of model decisions. The human evaluation scores are therefore interpreted as proxy indicators of spatial grounding rather than direct measurements of the model’s internal reasoning process [30]. Nevertheless, this framework reveals a major failure mode where accurate predictions are made without any significant meaningful spatial activation co-localization, a characteristic standard accuracy metrics cannot capture due to their inherent nature. The substantial improvement in answer justification consistency stood at 0.34 when compared with the model without PFCM and reached 0.46 relative to the variant excluding the SAAM. This demonstrates that spatially adaptive gating and progressive multi-scale fusion provide complementary grounding enhancements that no single module achieves in isolation. The quantitative bias assessment and these qualitative observations together position PMA-VQA as a robust framework for spatially grounded multi-scale visual question answering.

## 6. Conclusions

In this paper, we proposed PMA-VQA, a spatial-adaptive attention progressive multi-scale feature fusion framework for RS-VQA tasks. The core has two synergistic modules. While the SAAM performs language-conditioned gating at every stage of the backbone, the PFCM integrates high-level semantic abstractions and fine-grained geospatial detail. Evaluations on RS-VQA LR and RS-VQA HR demonstrated that PMA-VQA achieved superior overall and average accuracy relative to all compared fusion-based architectures and vision–language models, with the most notable improvements being in question types that demand fine-grained spatial discrimination. Supplementary evaluation on HRVQA, conducted under a city-level geographic partition with a 10-type question taxonomy, confirmed that both the SAAM and PFCM maintained their effectiveness under substantially different question-type distributions, with module-level attribution patterns consistent with those observed on RS-VQA. This verifies the efficacy of hierarchical, language-guided feature alignment. Further analysis of language bias evaluation strengthened the complementary debiasing potentials of the two modules. While the SAAM alleviates the overreliance on the statistical regularities of language, the PFCM maintains visual grounding with partially degraded inputs. This ensures stable relative accuracy across corruption families, resolution scales, and geographical domains. Future work will explore dynamic routing and the integration of ontology-based knowledge verification to further improve the interpretability and reliability of VQA systems in Earth observation applications.

## Figures and Tables

**Table 1:** Summary of notations and descriptions.

Symbol	Description
$I\in R^{3\times H\times W}$	Remote sensing image input
$Z_{0}\in R^{N_{0}\times d}$	Initial patch token sequence $N_{0}=\frac{H}{P}\times \frac{W}{P}$
$Z_{l}^{\left(i\right)}$	Output of the *l*th Swin block in stage *i*
$V_{i}\in R^{N_{i}\times d_{i}}$	Stage-*i* visual representation
$L\in R^{D\times N_{l}}$	Linguistic feature matrix (D=768)
$M_{L}\in {\left\{0,1\right\}}^{1\times N_{l}}$	Binary linguistic attention mask
$C_{i}\in R^{N_{i}\times d_{i}}$	SAAM cross-modal output at stage *i*
$\omega_{i}$	Position-specific spatial gating weight map at stage *i*
${\hat{V}}_{i}$	Gated residual visual representation
${\bar{C}}_{i}\in R^{d_{i}\times H_{i}\times W_{i}}$	Spatially mapped feature representation after layer normalization
$F_{\mathrm{fused}}$	Final concatenated multi-scale feature representation
$d_{i}$	Channel dimension at stage *i*: d,2d,4d,8d
*h*	Unified PFCM channel dimension (h=8d)

**Table 2:** Comparison of our PMA-VQA model with RS-VQA methods on RS-VQA LR test split. Bold indicates best result per column.

Methods	Count	Presence	Comparison	Rural or Urban	AA	OA
RS-VQA [6]	67.01	87.46	81.50	90.00	81.49	79.08
EasyToHard [13]	69.22	90.66	87.49	91.67	84.76	83.09
Bi-Modal [28]	72.22	91.06	91.16	92.66	86.78	85.56
SHRNet [29]	73.87	91.03	90.48	94.00	87.34	85.85
SAMFPN [7]	69.51	91.21	91.01	92.67	86.10	84.76
HFIF [14]	74.61	91.51	91.80	96.00	88.48	86.70
RSGPT [16]	–	91.17	91.70	94.00	–	–
SkyEyeGPT [17]	–	90.23	90.46	84.00	–	–
**PMA-VQA**	**76.04**	**91.71**	**92.27**	**97.00**	**89.25**	**87.38**

**Table 3:** Comparison of our PMA-VQA model with RS-VQA methods on RS-VQA HR Test 1 split. Bold indicates best result per column.

Methods	Count	Presence	Comparison	Area	AA	OA
RS-VQA [6]	68.63	90.43	88.19	85.24	83.12	83.23
EasyToHard [13]	69.06	91.39	89.75	85.92	83.97	84.16
Bi-Modal [28]	69.80	92.03	91.83	86.27	84.98	85.30
SHRNet [29]	70.04	92.45	91.68	86.35	85.13	85.39
SAMFPN [7]	69.19	91.99	91.44	86.83	84.61	84.94
HFIF [14]	70.46	**92.58**	91.95	86.44	85.36	85.69
RSGPT [16]	–	91.86	92.15	–	–	–
SkyEyeGPT [17]	–	87.59	88.63	–	–	–
**PMA-VQA**	**70.90**	92.27	**92.31**	**86.90**	**85.59**	**85.90**

**Table 4:** Comparison of our PMA-VQA model with RS-VQA methods on RS-VQA HR Test 2 split. Bold indicates best result per column.

Methods	Count	Presence	Comparison	Area	AA	OA
RS-VQA [6]	61.47	86.26	85.94	76.33	77.50	78.23
EasyToHard [13]	61.95	87.97	87.68	78.62	79.06	79.29
Bi-Modal [28]	63.06	89.37	89.62	80.12	80.54	81.23
SHRNet [29]	**63.42**	89.81	89.44	80.37	80.76	81.37
SAMFPN [7]	62.58	89.04	88.95	78.36	79.73	80.54
HFIF [14]	63.29	89.85	89.84	80.50	80.87	81.54
RSGPT [16]	–	**89.87**	89.68	–	–	–
SkyEyeGPT [17]	–	87.50	86.24	–	–	–
**PMA-VQA**	62.78	89.48	**90.36**	**80.94**	**80.89**	**81.55**

**Table 5:** Performance evaluation of PMA-VQA on HRVQA against GFTransformer across question categories. Trans. denotes transportation; Y/N denotes yes or no. ^†^ indicates results implemented using the authors’ released code. Bold indicates the best result per column.

Method	Per-Question-Type Accuracy (%)											Overall (%)	
	Shape	Color	Areas	Y/N	Numbers	Size	Location	Trans.	Scene	Sports		AA	OA
GFTransformer [27] ^†^	92.58	34.90	**99.75**	90.67	74.00	98.87	72.21	92.87	72.66	78.23		80.67	80.57
- w/o SAAM and PFCM	92.45	76.72	88.68	91.78	75.20	98.17	**78.68**	95.79	78.02	87.11		86.26	86.67
- w/o PFCM	93.05	76.67	87.13	92.87	76.98	98.87	73.29	**96.47**	76.66	**89.90**		86.19	87.47
- w/o SAAM	93.35	**78.09**	89.21	92.57	76.86	**98.91**	67.70	96.26	79.16	87.83		85.99	87.32
**PMA-VQA**	**94.26**	77.97	88.93	**93.34**	**77.13**	**98.91**	78.30	96.04	**79.34**	88.35		**87.26**	**88.36**

**Table 6:** Ablation study of the proposed SAAM and PFCM on the RS-VQA LR and RS-VQA HR datasets. ✓ and × denote the inclusion and exclusion of the corresponding module, respectively. Bold indicates best result per column.

Variant		RS-VQA LR		RS-VQA HR Test 1		RS-VQA HR Test 2	
SAAM	PFCM	AA	OA	AA	OA	AA	OA
×	×	83.06	83.49	83.99	84.25	79.28	79.49
×	✓	87.12	85.87	85.12	85.04	80.66	81.17
✓	×	87.75	86.33	85.26	85.37	80.23	81.25
✓	✓	**89.25**	**87.38**	**85.59**	**85.90**	**80.89**	**81.55**

**Table 7:** Fine-grained ablation of SAAM internal components on the RS-VQA LR dataset. “Cross-Attn” denotes the cross-modal spatial attention mechanism; “Spatial Gate” denotes the spatially adaptive gating; and “Residual Gate” denotes the residual gating connection. ✓ and × denote the inclusion and exclusion of the corresponding module, respectively. Bold indicates best result per column.

Cross-Attn	Spatial Gate	Residual Gate	Rural or Urban	Presence	Count	Comp	AA	OA
✓	×	×	93.00	91.24	72.83	91.33	87.12	85.87
✓	×	✓	95.00	90.52	74.79	91.88	88.05	86.47
✓	✓	×	**97.00**	91.47	75.53	91.82	88.95	86.97
✓	✓	✓	**97.00**	**91.71**	**76.04**	**92.27**	**89.25**	**87.38**

**Table 8:** Progressive multi-scale fusion ablation on the RS-VQA LR dataset. ${\bar{C}}_{i}$ denotes the cross-modal feature map from the SAAM at stage *i*. Feature levels were incrementally incorporated from abstract semantic representations to fine-grained spatial details. ✓ indicates the corresponding feature level is included; empty cells represent exclusion. Bold indicates best result per column.

Semantic Level			Spatial Level			Question Type Accuracy (%)					Overall (%)	
${\bar{C}}_{4}$	${\bar{C}}_{3}$		${\bar{C}}_{2}$	${\bar{C}}_{1}$		**Count**	**Presence**	**Comparison**	**Rural or Urban**		**AA**	**OA**
✓						73.77	91.51	91.55	**99.00**		88.96	86.38
✓	✓					74.65	91.44	91.78	95.00		88.22	86.67
✓	✓		✓			74.99	**92.05**	91.85	96.00		88.72	86.99
✓	✓		✓	✓		**76.04**	91.71	**92.27**	97.00		**89.25**	**87.38**

**Table 9:** Human evaluation of Grad-CAM spatial grounding on 50 randomly sampled RS-VQA LR test instances. Scores ranged from 0 to 2 (higher is better). SRS = spatial relevance score; AJC = answer justification consistency; CS = context sensitivity. Bold indicates best result per column.

Model	SRS	AJC	CS
w/o SAAM	1.24	1.20	1.10
w/o PFCM	1.16	1.32	1.18
PMA-VQA	**1.50**	**1.66**	**1.46**

**Table 10:** Overall bias evaluation across two RS-VQA datasets. The table compares the overall accuracy (OA), adversarial testing (AdTest), and relative accuracy (RelAcc) of our full PMA-VQA model against its ablated variants (w/o SAAM and w/o PFCM) under black (*B*), white (*W*), and noise (*N*) adversarial strategies. Bold indicates best result per column.

Dataset	Method	OA	Black (*B*)			White (*W*)			Noise (*N*)	
		(%)	AdTest	RelAcc		AdTest	RelAcc		AdTest	RelAcc
**RS-VQA LR**	w/o SAAM	85.87	53.71	69.48		52.98	69.95		50.88	71.23
	w/o PFCM	86.33	51.33	71.91		50.89	72.16		49.36	73.01
	**PMA-VQA**	**87.38**	49.22	**75.15**		48.77	**75.37**		49.65	**74.94**
**RS-VQA HR Test 1**	w/o SAAM	85.04	60.68	61.95		61.43	61.21		63.08	59.48
	w/o PFCM	85.37	60.17	63.27		61.46	62.04		62.82	60.65
	**PMA-VQA**	**85.90**	60.76	**64.07**		61.98	**62.91**		62.19	**62.71**
**RS-VQA HR Test 2**	w/o SAAM	81.17	57.42	55.78		58.45	54.68		58.38	54.76
	w/o PFCM	81.25	56.98	56.42		58.19	55.15		58.70	54.60
	**PMA-VQA**	**81.55**	56.43	**57.66**		57.23	**56.86**		59.06	**54.93**

**Table 11:** Computational cost across PMA-VQA ablation configurations. Params = trainable parameters (M); FLOPs = per-sample floating-point operations (G); Mem = peak GPU memory (GB); Throughput = samples/s. ✓ and × denote the inclusion and exclusion of the corresponding module, respectively.

Variant		Params	FLOPs	GPU Mem	Throughput
SAAM	PFCM				
×	×	95.42	47.15	1.034	42.01
×	✓	99.07	48.08	1.047	42.90
✓	×	100.99	49.57	1.043	40.63
✓	✓	104.64	50.50	1.058	41.07

**Figure A1:**
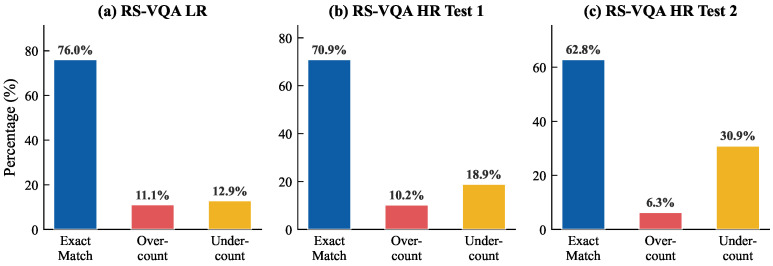
Error direction breakdown of PMA-VQA counting predictions across the three evaluation splits.

**Figure A3:**
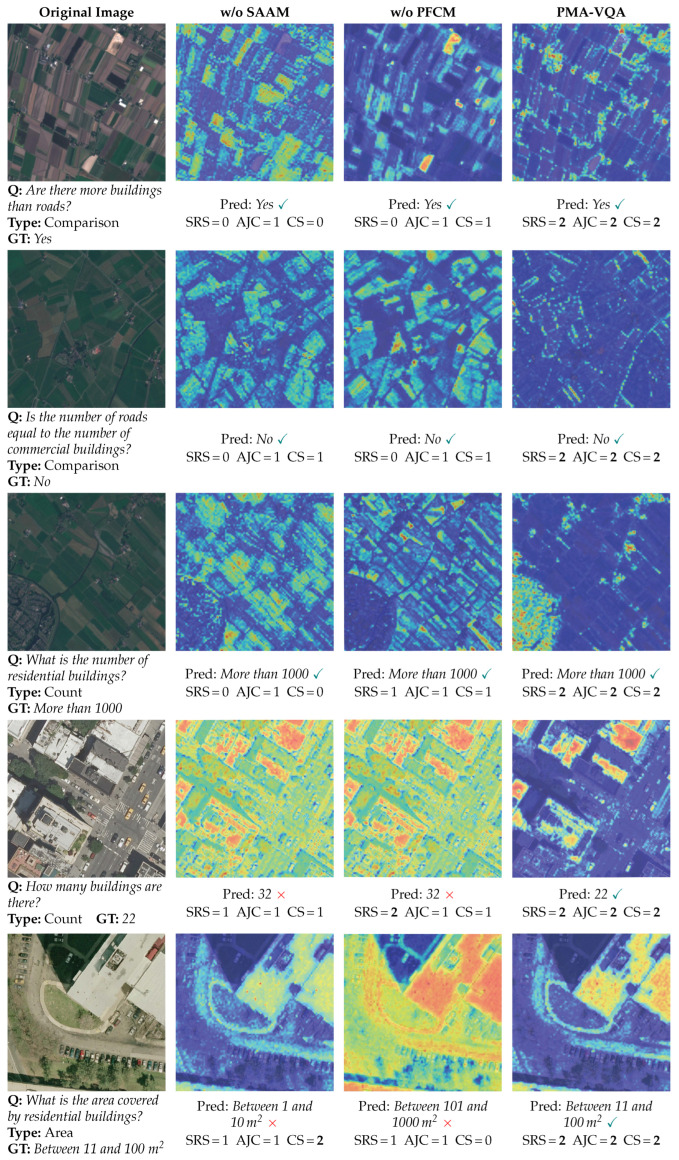
Human evaluation scores from 0 to 2 across five representative test instances from RS-VQA LR (Cases 1–3) and RS-VQA HR (Cases 4–5) under three model configurations. Each cell reports the predicted answer, with ✓ and × denoting correct and incorrect predictions, respectively, along with the spatial relevance score, answer justification consistency, and context sensitivity.

## Data Availability

The code for PMA-VQA is available at https://github.com/Oscarnibaba/PMA-VQA.git (accessed on 6 April 2026).

## Appendix A. Per-Category Language Bias Analysis

Table A1 further decomposes the bias indicators by question type. The RS-VQA LR dataset showed the strongest debiasing impact of PMA-VQA for the “Rural or Urban” queries, with the RelAcc hitting 93.18% consistently for all three corruption settings. These values surpassed the SAAM- and PFCM-ablated variants by 9.09 and 6.82 percentage points, respectively. This suggests that the full model learned truly discriminative visual features for land use classification rather than exploiting language priors. The same pattern can be seen in the “Comparison” queries, with PMA-VQA showing RelAcc improvements over the ablated variants of up to 6.00 percentage points. Regarding the “Presence” queries, during black and white conditions, the PMA-VQA led, while the SAAM-ablated variant scored slightly better under noise (79.63% vs. 78.43%), indicating that spatial gating may slightly redistribute attention away from binary existence decisions in the presence of noise. Overall, the full model maintained the most consistent debiasing performance across question types and corruption families.

**Table A1:** Detailed bias and ablation analysis on the RS-VQA LR and HR Test Set 1 and HR Test Set 2. The OA and RelAcc are compared for black (*B*), white (*W*), and noise (*N*) settings. The highest RelAcc and OA in each question type are highlighted in bold to indicate the strongest performance.

Dataset	OA (%)				Black (*B*) RelAcc				White (*W*) RelAcc				Noise (*N*) RelAcc		
	w/o SAAM	w/o PFCM	PMA-VQA		w/o SAAM	w/o PFCM	PMA-VQA		w/o SAAM	w/o PFCM	PMA-VQA		w/o SAAM	w/o PFCM	PMA-VQA
**RS-VQA LR Dataset**															
Comparison	91.33	91.18	**92.27**		78.15	79.42	**82.91**		77.70	80.49	**83.60**		77.53	81.35	**83.53**
Count	72.83	74.48	**76.04**		59.02	62.58	**64.23**		59.87	62.53	**64.20**		61.13	62.24	**64.32**
Presence	91.24	91.30	**91.71**		75.46	78.43	**80.43**		79.02	78.33	**79.97**		**79.63**	77.41	78.43
Rural/Urban	93.00	94.00	**97.00**		84.09	86.36	**93.18**		84.09	86.36	**93.18**		84.09	86.36	**93.18**
**LR Overall**	85.87	86.33	**87.38**		69.48	71.91	**75.15**		69.95	72.16	**75.37**		71.23	73.01	**74.94**
**RS-VQA HR Test Set 1**															
Area	86.59	86.78	**86.90**		**75.01**	66.28	74.24		**73.11**	66.43	71.72		62.85	66.09	**66.90**
Comparison	91.68	91.66	**92.31**		77.10	**79.39**	77.14		76.30	**78.87**	77.67		77.37	77.07	**79.28**
Count	70.33	70.54	**70.90**		**28.51**	26.84	26.92		**29.49**	26.24	27.10		**28.09**	25.70	26.92
Presence	91.88	92.06	**92.27**		78.04	**79.62**	78.10		78.15	77.65	**78.53**		77.04	77.14	**78.30**
**HR1 Overall**	85.04	85.37	**85.90**		61.95	63.27	**64.07**		61.21	62.04	**62.91**		59.48	60.65	**62.71**
**RS-VQA HR Test Set 2**															
Area	80.68	80.88	**80.94**		**65.57**	58.44	65.26		58.90	58.35	**61.44**		57.81	**58.19**	57.10
Comparison	89.29	89.95	**90.36**		70.56	**77.06**	75.23		73.39	74.93	**76.65**		72.81	74.36	**75.32**
Count	62.34	61.86	**62.78**		**19.86**	14.46	15.00		**17.95**	14.22	15.06		**16.18**	14.06	14.24
Presence	89.23	89.03	**89.48**		72.28	74.25	**75.47**		72.79	71.89	**72.96**		72.23	71.44	**73.64**
**HR2 Overall**	81.17	81.25	**81.55**		55.78	56.42	**57.66**		54.68	55.15	**56.86**		54.76	54.60	**54.93**

On the RS-VQA HR Test Set 1, the per-category results further illuminate the contribution of each module. PMA-VQA exceeded both ablated variants under noise corruption (66.90% vs. 66.09% and 62.85%), showing that its benefit manifested when the visual signal was degraded only partially. For HR Test Set 2, PMA-VQA showed the greatest visual dependence on “Area” queries on black (65.26%) and white (61.44%) conditions and “Comparison” queries on noise (75.32%). The RelAcc values for the full model show stability for the presence category at 72–75%. This shows that object existence reasoning transfers reasonably well across geographical domains.

All variants of the model found the “Counting” category to be the most difficult one, where the RelAcc values were between 25% and 29% on the RS-VQA HR Test 1. This illustrates the particular and considerable language bias when there were numerical responses in high-resolution scene contexts. PMA-VQA reached the highest counting OA on the RS-VQA LR dataset, achieving 76.04%. It also exhibited the narrowest RelAcc fluctuation of only 0.12 percentage points (64.20–64.32%) across all three adversarial scenarios. In contrast, the SAAM-ablated variant had a range of 2.11 percentage points (59.02–61.13%), and for the PFCM-ablated variant, it was 0.34 percentage points (62.24–62.58%). The results show that when the SAAM and PFCM were deployed together, there was greater counting behavior accuracy and stability in comparison with the other configured alternatives assessed.

## Appendix B. Training Stability Analysis

In Table A2, we conducted two additional independent training runs with distinct random seeds. The consistently low standard deviations across all splits and question types confirm that PMA-VQA converged stably, and the reported performance gains were not attributable to fortuitous initialization. On the RS-VQA LR dataset, the OA standard deviation over three runs was only 0.05%, and the AA standard deviation was 0.03%. In particular, the “Rural or Urban” class obtained a standard deviation of 0.00%, signifying completely deterministic scene-level classification behavior across seeds. For HR Test Set 1, the OA standard deviation was 0.02% while the AA was 0.04%. The slightly larger standard deviation for the “Area” category (0.21%) reflects a sensitivity to the spatial sampling variability inherent in high-resolution scenes, as opposed to variability in the training. On HR Test Set 2, all per-category standard deviations remained at or below 0.08%, and the OA standard deviation was 0.03%. Taken together, these results confirm that PMA-VQA exhibited robust and reproducible generalization across random seeds, training configurations, and geographical domains.

**Table A2:** Training stability of PMA-VQA across three independent runs. Run 1 corresponds to the result reported in the main comparison tables. Mean and standard deviation (Std) were computed over the three runs. AA = average accuracy; OA = overall accuracy. Bold indicates best result per column.

Dataset	Run	Count	Presence	Comparison	Rural/Urban	AA (%)	OA (%)
**RS-VQA LR**	Run 1	76.04	91.71	92.27	97.00	89.25	87.38
	Run 2	75.88	91.71	92.15	97.00	89.19	87.28
	Run 3	75.88	91.84	92.15	97.00	89.22	87.33
	Mean	75.93	91.75	92.19	97.00	89.22	87.33
	Std	±0.09	±0.08	±0.07	±0.00	±0.03	±0.05
**RS-VQA HR Test 1**		**Count**	**Presence**	**Comparison**	**Area**	**AA (%)**	**OA (%)**
	Run 1	70.90	92.27	92.31	86.90	85.59	85.90
	Run 2	70.94	92.35	92.27	86.53	85.52	85.87
	Run 3	70.81	92.25	92.33	86.55	85.52	85.86
	Mean	70.88	92.29	92.30	86.66	85.54	85.88
	Std	±0.07	±0.05	±0.03	±0.21	±0.04	±0.02
**RS-VQA HR Test 2**		**Count**	**Presence**	**Comparison**	**Area**	**AA (%)**	**OA (%)**
	Run 1	62.78	89.48	90.36	80.94	80.89	81.55
	Run 2	62.63	89.36	90.25	80.84	80.77	81.50
	Run 3	62.70	89.42	90.30	80.88	80.83	81.52
	Mean	62.70	89.42	90.30	80.89	80.83	81.52
	Std	±0.08	±0.06	±0.06	±0.05	±0.06	±0.03

## Appendix C. Supplementary Counting Error Distributions

Figure A1 decomposes the counting predictions into exact matches, overcounts, and undercounts across the three evaluation splits, visually confirming the conservative undercounting tendency reported in Section 4.8. Figure A2 presents the full signed prediction error distribution (Pred−GT) for the two HR test sets, illustrating that the error mass was sharply concentrated around zero on both splits, despite the pronounced shift in the mean signed error.

## Appendix D. Human Evaluation: Extended Protocol and Case Study

Table A3 augments the mean scores reported in Table 9 with the Top 2 rate, defined as the proportion of samples for which the mean annotator score equals two. PMA-VQA attained unambiguously high ratings across all three criteria, with a Top 2 rate of 56% for spatial relevance, 70% for answer grounding, and 50% for context discrimination, consistently surpassing both ablated variants.

**Table A3:** Extended human evaluation results. Mean score (0–2) and Top 2 rate (%) per criterion and model variant over 50 RS-VQA LR test instances. Bold indicates best per column.

Model	Mean Score (0–2)				Top 2 Rate (%)		
	SRS	AJC	CS		SRS	AJC	CS
w/o SAAM	1.24	1.20	1.10		34.0	38.0	20.0
w/o PFCM	1.16	1.32	1.18		26.0	42.0	24.0
PMA-VQA	**1.50**	**1.66**	**1.46**		**56.0**	**70.0**	**50.0**

The per-criterion breakdown revealed a module-specific attribution pattern. The “w/o PFCM” variant retained a higher AJC mean of 1.32 compared with 1.20 for “w/o SAAM”, indicating that the SAAM’s per-position linguistic gating is the primary contributor to answer-grounding consistency. Conversely, “w/o SAAM” yielded a higher SRS mean of 1.24 compared with 1.16 for “w/o PFCM”, confirming that the PFCM’s multi-resolution context is the principal driver of attention co-localization with query-relevant objects. The joint deployment of both modules in PMA-VQA yielded the highest scores across all criteria, demonstrating that the two modules contributed complementary rather than redundant functional improvements.

Figure A3 presents five representative instances spanning question types, dataset splits, and prediction outcomes. Cases 1–3 were from RS-VQA LR, and Cases 4–5 were from RS-VQA HR.

In Cases 1 and 2, all three variants predicted the correct answer, yet both ablations scored an SRS of zero, indicating that correct predictions were driven by linguistic priors rather than spatial evidence. PMA-VQA achieved an SRS of two, an AJC of two, and a CS of two in both instances, demonstrating that prediction accuracy alone was an insufficient indicator of genuine visual grounding. Case 3 extended this finding to a dense low-resolution scene, where “w/o SAAM” scored an SRS of zero and a CS of zero, reflecting uniform activation, while “w/o PFCM” improved to an SRS of one and a CS of one, confirming that linguistic gating alone is insufficient to capture the distributional extent of small, dense objects without multi-scale feature support.

Case 4 shows that both ablations overcounted by 10 instances, predicting 32 against a ground truth of 22. The “w/o PFCM” variant correctly localized the building cluster with an SRS of two yet still miscounted with an AJC of one, establishing that spatial co-localization is necessary but insufficient for accurate enumeration without scale-aware boundary resolution. Case 5 exposed a bidirectional failure pattern; “w/o SAAM” underestimated the area with an SRS of one and a CS of two, indicating that without linguistic gating, the model attended to the correct general region yet systematically underweighted the spatial extent of residential footprints. Meanwhile, “w/o PFCM” overestimated the area with an SRS of one and a CS of zero, indicating that reliance on a single feature scale caused the model to conflate target footprints with adjacent land cover boundaries. PMA-VQA attained correct predictions with an SRS of two in both instances, corroborating that precise area estimation necessitates the simultaneous functioning of spatially adaptive gating and progressive multi-scale fusion.
